# Impact of Genetic Factors on the Age of Onset for Type 2 Diabetes Mellitus in Addition to the Conventional Risk Factors

**DOI:** 10.3390/jpm11010006

**Published:** 2020-12-22

**Authors:** Peter Piko, Nardos Abebe Werissa, Szilvia Fiatal, Janos Sandor, Roza Adany

**Affiliations:** 1 MTA-DE Public Health Research Group, University of Debrecen, 4028 Debrecen, Hungary; piko.peter@med.unideb.hu (P.P.); nardos.abebe@med.unideb.hu (N.A.W.); 2 Doctoral School of Health Sciences, University of Debrecen, 4032 Debrecen, Hungary; 3 Department of Public Health and Epidemiology, Faculty of Medicine, University of Debrecen, 4032 Debrecen, Hungary; fiatal.szilvia@med.unideb.hu (S.F.); sandor.janos@med.unideb.hu (J.S.)

**Keywords:** age of onset for type 2 diabetes, single nucleotide polymorphism, genetic risk score, type 2 diabetes mellitus, Hungarian population

## Abstract

It is generally accepted that the early detection of type 2 diabetes mellitus (T2DM) is important to prevent the development of complications and comorbidities, as well as premature death. The onset of type 2 diabetes mellitus results from a complex interplay between genetic, environmental, and lifestyle risk factors. Our study aims to evaluate the joint effect of T2DM associated single nucleotide polymorphisms (SNPs) on the age of onset for T2DM in combination with conventional risk factors (such as sex, body mass index (BMI), and TG/HDL-C ratio) in the Hungarian population. This study includes 881 T2DM patients (Case population) and 1415 samples from the Hungarian general population (HG). Twenty-three SNPs were tested on how they are associated with the age of onset for T2DM in the Case population and 12 of them with a certified effect on the age of T2DM onset were chosen for an optimized genetic risk score (GRS) analysis. Testing the validity of the GRS model developed was carried out on the HG population. The GRS showed a significant association with the age of onset for T2DM (β = −0.454, *p* = 0.001) in the Case population, as well as among T2DM patients in the HG one (β = −0.999, *p* = 0.003) in the replication study. The higher the GRS, the earlier was the T2DM onset. Individuals with more than eight risk alleles will presumably be diabetic six and a half years earlier than those with less than four risk alleles. Our results suggest that there is a considerable genetic predisposition for the early onset of T2DM; therefore, in addition to conventional risk factors, GRS can be used as a tool for estimating the risk of the earlier onset of T2DM and stratifying populations at risk in order to define preventive interventions.

## 1. Introduction

The global prevalence of diabetes among adults rose from 4.7% to 8.5% between 1980 and 2014 [1]. The majority of diabetic patients (~90%) have type 2 diabetes mellitus (T2DM) [2]. Previously, T2DM was considered to be a disease of older adults (formerly known as adult-onset diabetes); however, in recent decades the age of onset has been falling and T2DM has been reported in younger adults and even in children and adolescents as well [3,4]. In addition to the growth of the proportion of younger age groups among T2DM patients, the emergence of it is still characteristic of late middle-aged and older people. The highest estimated crude incidence of diagnosed diabetes was among people aged 45–64 years in the United States of America in 2018 [5]. This number was lower in those under 45 or over 64 years [6].

Early onset T2DM (EOT2DM), defined as a diagnosis at the age below 45 years of age, has become more prevalent throughout the world since the early 1990s [7]. Several countries around the world (e.g.,: China [8], the United Kingdom [9]), reported an increase in the incidence and consequently the prevalence of EOT2DM. In Japan [10] and Taiwan [11], more than 50% of diabetes cases in children and adolescents have type 2 diabetes mellitus.

The early onset of T2DM exerts an extensive impact on individuals, healthcare service planning, and delivery [7]. It is supported by evidence that individuals with EOT2DM appear to have poorer quality of health outcomes compared with the non-diabetic population or individuals with the usual-onset of T2DM (UOT2DM) [12,13]. It was also corroborated that EOT2DM is associated with a higher risk of many types of diabetes-related complications [12,14]. The odds of developing macrovascular and microvascular complications are higher among diabetes patients with EOT2DM compared with UOT2DM [12]. Individuals with EOT2DM have a much higher risk of the development of cardiovascular diseases, and microalbuminuria is also more common among them compared with people with UOT2DM [15]. In general, individuals with EOT2DM have significantly poorer metabolic profiles than individuals with UOT2DM [16].

Diabetes was the seventh leading cause of death estimated by the WHO in 2016 (it was 13th in 2000) [17]. The early identification of T2DM could help reduce the number of deaths caused by them [18]. To develop effective screening, it is essential to know the key factors involved in the development of the disease. Type 2 diabetes mellitus results from a complex interplay between multiple genetic factors and a wide variety of environmental and lifestyle risk factors, such as physical inactivity, obesity, high sugar intake, and low socio-economic status [7]. It is a well-known fact that genetic factors also play a major role in the development of diabetes (the estimated heritability of fasting blood sugar is 29%) [19]. However, until now, only a small number of studies have focused on how these genetic factors contribute to the age of the development of T2DM. The existence of genetic causes underlying EOT2DM is supported by the fact that in one study 81% of children and young people had a family history of T2DM (70% with first-degree relatives: 17% both parents affected, 50% mother alone, 23% father alone, and 10% sibling alone affected, and 11% with second-degree relatives) in the United Kingdom [20]. Over the last two decades, several single nucleotide polymorphisms (SNPs) have been shown to be associated with the development of EOT2DM [21,22,23,24,25,26,27,28,29,30]. The majority of these SNPs exert their effect on disease risk through deficient insulin secretion [31].

In Hungary, according to the 2019 International Diabetic Federation report, the age-adjusted comparative prevalence of diabetes (type 1 and 2) among the adult population (aged between 20–70 years) is 6.9%, more than 90% of which is accounted for by T2DM [32]. A recent nationwide population-based study showed that the prevalence of T2DM among children and adolescents in Hungary was 22 out of 100,000 [33] and this data was higher than in most European countries (such as Denmark [34] and Germany [35]). The prevalence of obesity among children or adolescents was 6.6% [36], while among adults (above 18 years) it was 32% [37]. Hungary has the highest obesity rate in Europe based on OECD data from 2016 [38]. Although the high incidence of obesity in the Hungarian population can be partly explained by the high incidence of T2DM, the underlying genetic causes cannot be neglected either [39,40].

Our previous studies confirmed that in the case of obesity [39,41] and T2DM [40], in addition to environmental and lifestyle factors, genetic susceptibility is also very significant in the Hungarian general population. T2DM is developing earlier for individuals with a higher genetic risk, in comparison with people with the same lifestyle and environmental characteristics but with a lower genetic risk [42]. By identifying these genetically high-risk individuals, it is possible to develop a cost-effective targeted prevention program for EOT2DM. So far only a limited number of studies have examined the effect of genetic factors (such as SNPs) on the age of development of T2DM, and most of these have been studied in Asian, as Chinese Han, Japanese and Taiwanese populations [31,43,44,45]. Although in a genome-wide search for type 2 diabetes-related traits in a group of people with French ancestry evidence for a susceptibility locus for diabetes and impaired glucose tolerance with early onset (at age < 46 years) on chromosome 3q27-qter was provided [46], we have little knowledge (restricted to diabetics in Tayside, Scotland) on European populations [47].

Our current research aims to determine whether the addition of genetic risk variant (SNPs) data related to the development of T2DM in the form of genetic risk scores to conventional risk factors (sex, BMI, TG/HDL-C ratio) improves risk assessment of the early onset of T2DM or not. Furthermore, based on the relevant SNPs identified in the T2DM case population, a genetic risk score model was created and tested on an independent sample population (representative of the Hungarian general population). The contribution of genetic and non-genetic factors to the development of T2DM was also tested on patients with EOT2DM and UOT2DM.

## 2. Materials and Methods

### 2.1. Sample Populations

#### 2.1.1. T2DM Case Population Representative of Hungarian T2DM Patients above 50 Years Old

The study subjects were obtained from a previous survey (in 2008), which was conducted in the framework of General Practitioner’s Morbidity Sentinel Stations Program and included 1168 individuals representative of Hungarian T2DM patients above the age of 50 years [48]. Physical examinations (weight, height, waist circumference, and blood pressure) were carried out and blood samples (native and EDTA-anticoagulated) for laboratory investigation and DNA isolation were collected by physicians. Information on sociodemographic characteristics and self-assessed health status were obtained using a self-administered questionnaire. More details on sample collection are described by Nagy et al. [49].

#### 2.1.2. Hungarian General Population

One-thousand seven-hundred and eighty-three samples from the Hungarian general population, obtained from our previous study from 2008 [50], were included and used as an independent population for validating the genetic risk score model. The Hungarian general sample population is representative for the Hungarian adult (>20 years) population in terms of age, sex, and geographic distribution. As part of the survey, interviewer-assisted questionnaires were used to collect data on sociodemographic factors and self-assessed health status. Medical histories were recorded, and each participant went through physical examination. Native and EDTA-anticoagulated blood samples were taken for genotyping and laboratory examinations. Further details of sample collection and characteristics are described by Szigethy et al. and Kosa et al. [50,51].

### 2.2. DNA Extraction, Selection of SNPs, Genotyping, Testing Hardy-Weinberg Equilibrium and Linkage Disequilibrium

DNA was extracted from EDTA-anticoagulated blood samples using the DNA Isolation Kit–Large Volume on a MagNA Pure LC instrument (Roche Diagnostics, Basel, Switzerland) following the manufacturer’s instructions.

Twenty-three SNPs that affect the development of T2DM were identified through a systematic literature search on online databases (PubMed, HuGE Navigator, and Ensembl). Details on the selection process of SNPs was described by Werissa et al. [40] and showed also in Table S1.

Genotyping was performed on the MassARRAY platform (Sequenom Inc., San Diego, CA, USA) with iPLEX Gold chemistry by the service provider (Mutation Analysis Core Facility of the Karolinska University Hospital (MAF), Sweden). Validation, concordance analysis, and quality control were conducted by the MAF, according to their protocols.

A χ^2^ test was used to assess whether the agreement of frequencies of genotypes for SNPs with Hardy-Weinberg equilibrium (HWE) expectations (by Plink software [52]). Linkage disequilibrium (LD) between polymorphisms was tested by Haploview software (version 4.2). In the presence of an LD block, one SNP was selected from each block to avoid multicollinearity.

### 2.3. Sample Selection for Study Populations

For T2DM Case and Hungarian general populations those samples were selected that have a complete record of diabetes-related pheno- and genotype data. These data include age, sex, body mass index (BMI), triglyceride (TG), and high-density lipoprotein cholesterol (HDL-C) levels in both populations and the age at diagnosis of diabetes in the T2DM case population (the age at diagnosis is not available for subjects of the Hungarian general one). The TG to HDL-C ratio (TG/HDL-C ratio) was defined as a better indicator for T2DM [53] than TG or HDL-C levels individually.

The samples of the HG population were divided into three subpopulations based on their fasting glucose levels (FG) and/or treatment for diabetes.

The three subpopulations:

1. Subjects with normal fasting glucose level: FG < 5.6 mmol/L, *n* = 1197
2. Prediabetic subjects: FG between 5.6 and 6.9 mmol/L, *n* = 108
3. Type 2 diabetic patients: any person who had a FG level of 7 mmol/L or higher and/or was under antidiabetic treatment, *n* = 110 [54]

### 2.4. Determination of the Best Fitting Genetic Model for SNPs

For each SNP we tested [55] which of the genetic model of inheritance (codominant, dominant, and recessive) showed the strongest correlation with the outcome (age of onset for T2DM) in the Case population. Adjusted (by age, sex, TG/HDL-C ratio) regression analyses were applied to test the association of SNPs individually with the age of onset for T2DM by SNPStats online tool (http://bioinfo.iconcologia.net/SNPstats). The Akaike information criterion (AIC), Bayesian information criterion (BIC), and *p*-value were used to find the best fitting genetic model of inheritance under the selection process [56].

### 2.5. Unweighted Genetic Risk Score Calculation

#### 2.5.1. Coding SNPs by the Best Fitting Genetic Model

The best fitting genetic model of inheritance was determined for each SNP and these SNPs were encoded according to the criteria of the model as follows:

1. In case of the codominant genetic model:
  homozygote genes with two risk alleles were counted as “2′’, while heterozygote genotypes as “1′’ and homozygote non-risk genes as “0′’.
2. In case of the dominant genetic model:
  homo- and heterozygote genes with two or one risk alleles were counted as “2′’, while homozygote non-risk genes as “0′’.
3. In case of the recessive genetic model:
  homozygote genes with two risk alleles were counted as “2′’, while heterozygote genotypes with one risk allele and homozygote without risk allele as “0′’.

#### 2.5.2. Calculation and Optimization of the GRS Model

Subsequently, the number of risk effects (2, 1, or 0) was summed using equation 1, where Gi is the number of risk effects in the respective locus (see more detailed in Section 2.5.1) for the unweighted genetic risk score.

(1) $$GRS=\overset{I}{\underset{i=1}{\sum }}G_{i}$$

The effect of SNPs and the strength of their association on the age of onset for T2DM were determined as described in Section 2.4.

During the optimization of the GRS model, SNPs which do not reinforce the association of the model with the outcome variable were excluded. To avoid the possibility of false-positive association, SNPs were tested in an ascending order of *p*-value (from the strongest association with the weakest one). Starting with the SNP with the lowest *p*-value, we inserted them one by one in GRS model, the association of which with the age of onset was tested after each inserted SNP. For each step, the number of risk alleles for the next SNP was added to the GRS. Regression analysis was applied to monitor the changes in the strength of the association. The inserted SNPs were selected for the final GRS model only if they increased the model’s R-squared value and decreased its *p*-value.

Under the optimization process, all calculation was adjusted by BMI, TG/HDL-C ratio, sex, and duration of T2DM.

### 2.6. Estimation of the Effect of Genetic (Unweighted Genetic Risk Score) and Non-Genetic (Sex, BMI, and TG/HDL Ratio) Factors on the Age of Onset for T2DM on the Case Population

Linear regression was used to estimate the effect of GRS and non-genetic factors (sex, BMI, and TG/HDL ratio) on the age of onset for T2DM on the Case population. The results of this calculation were used to determine the weighted GRS as well as to construct a risk estimation model for the age of onset for T2DM on the HG population.

### 2.7. Weighted Genetic Risk Score Calculation

Weighted GRS calculation was performed on the HG population by using the beta values determined on the Case population for weighting (wβ_i). Then the GRS for each person (xi) was multiplied by the weight (wβ_i). Equation (2) describes the calculation of the weighted genetic risk score.

(2) $$wGRS=\overset{I}{\underset{i=1}{\sum }}w_{\beta \_i}X_{i}$$

### 2.8. Calculation of a Score for an Estimated Age of Onset for T2DM

The weight of genetic and non-genetic factors was determined by the Case population. Using these weights, it is possible to calculate a score to estimate the age of onset for T2DM. To investigate the combined effect of non-genetic (sex, BMI, and TG/HDL-C ratio) and genetic (GRSs) factors with a reasonable impact on the development of T2DM, a score was calculated for each sample. The effect of non-genetic and genetic factors on the age of onset for T2DM was estimated on the Case population and it was tested on the HG one.

### 2.9. Statistical Analysis

The normality of data for quantitative variables was tested using the Shapiro-Wilk test; and when it was necessary, non-normal variables were transformed using Templeton’s two-step approach [57]. Two-tailed Student’s *t*-tests were used to assess the statistical difference of variables among the groups. Multiple linear regression analyses were used to estimate the individual and combined (GRS) effect of SNPs on the early onset of T2DM. The Jonckheere-Terpstra trend test was [58] used to analyze the statistically significant trend between the ordinal independent variable and continuous or ordinal dependent variables.

All biostatistics calculations were adjusted by relevant covariates (e.g., sex, BMI, and TG/HDL-C ratio). IBM SPSS statistics for Windows (version 26, IBM Company, Armonk, NY, USA) and the SNPStats online tool were employed to carry out regression analyses. The Bonferroni correction was applied when several statistical tests were being performed simultaneously (*p* < 0.0042).

### 2.10. Ethical Statement

All subjects gave their informed consent to participate in the study. The study was conducted following the ethical standards of the institutional and national research committees, in harmony with the declaration of Helsinki. This study was commenced after getting ethical permission from the Ethical Committee of the University of Debrecen, Medical Health Sciences Centre (reference No. 2699-2007), and by the Ethical Committee of the Hungarian Scientific Council on Health (reference No. TUKEB 48495-2/2014/EKU).

## 3. Results

### 3.1. Characteristics of the Study Populations

**Figure 1:**
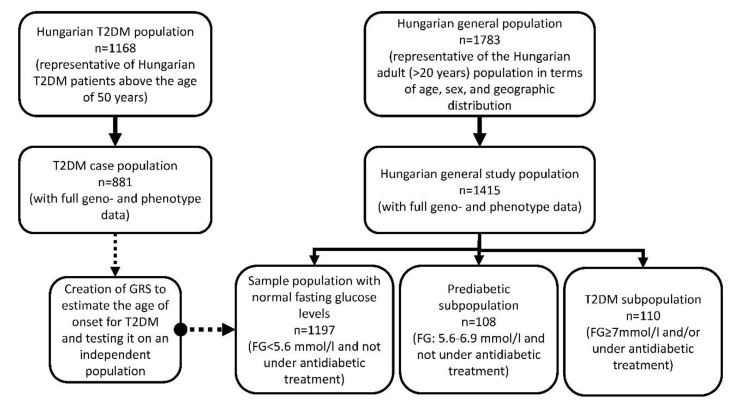
Flowchart showing the processes of sample selection, the stratification of the HG population, and the development and use of the GRS instrument.

Samples without full geno- and phenotype data were excluded from the analyses. In total, 881 individuals (males: 434, females 447) from the Case and 1415 (males: 669, females: 746) from the HG population were included. Three subpopulations were created from the HG one based on their fasting glucose levels and the presence of antidiabetic treatment. For more details on the process of sample selection and the creation of the study populations see Figure 1.

The population characteristics of the Case population was similar to the prediabetic and T2DM subpopulations of the HG and significantly differed from the subpopulation with a normal fasting glucose level. A statistically significant increase was observed in the proportion of males, and in the average age, BMI, TG levels, and TG/HDL-C ratio in case of samples ranging from normal FG levels to T2DM cases in the HG population, while HDL-C levels significantly decrease in the subpopulations. The age and gender differences between the Case and the Hungarian T2DM subpopulations are due to the age category (20–64 years) applied in the sample collection of the Hungarian general population. Details on population characteristics are listed in Table 1.

### 3.2. Results of the Hardy-Weinberg Equilibrium and Linkage Disequilibrium Analysis in the Case Population

**Figure 2:**
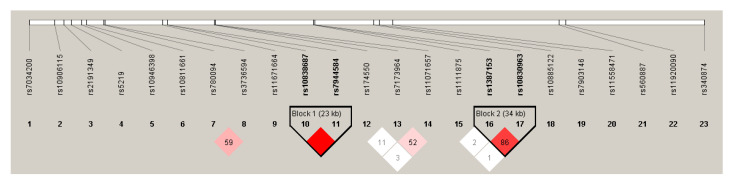
Linkage disequilibrium map of observed SNPs in the T2DM case population.

Regarding genotype distribution, no significant deviation from HWE was found in the Case population. Two blocks were identified within linkage disequilibrium (LD) (Block 1: rs10838687 and rs7944584; Block 2: rs1387153 and rs10830963). To avoid multicollinearity, only one SNP per LD block was used in the GRS calculation. In both cases, the first SNP of the LD block (rs1083687 and rs1387153) was excluded from further analyses. The results of the LD analysis can be seen in a more detailed form in Figure 2.

### 3.3. Results Obtained from the Analysis of the Determination of the Best Fitting Genetic Model for SNPs in the Case Population

Adjusted (by BMI, TG/HDL-C ratio, sex, and duration of T2DM) linear regression analyses were used to test the association of SNPs with the age of onset for T2DM in the Case population. For each SNP, we tested which of the three most commonly used genetic models of inheritance (codominant, recessive, and dominant) shows the strongest correlation with the age of onset for T2DM. The model with the lowest AIC, BIC, and *p*-value was chosen for genetic risk calculation. In 15 cases in the recessive, in five cases in the dominant, and in one case in the codominant model SNPs showed the strongest correlation with the age of onset. For the more detailed results of the calculation see Table S1.

### 3.4. Results of the Calculation and Optimization of the GRS Model

In calculating the GRS, we sought to select those SNPs that strengthen the association of the GRS with the outcome (age of onset for T2DM) in the linear regression model. We moved from the SNP with the strongest correlation (rs174550; beta = −0.866, *p* = 0.073) to the weakest (rs2191349; beta = −0.004, *p* = 0.990). The SNPs were individually inserted and tested by adjusted (by BMI, TG/HDL-C, sex, and duration of T2DM) linear regression models. All SNPs that strengthened the GRS association with the outcome variable (raised the value of R-squared) were selected and inserted in the final model of GRS, while those that weakened (reduced the value of R-squared) were excluded from it. During the selection process, 12 SNPs were selected for the final GRS model. For more details on the result of GRS optimization process see Table S2.

The 12 SNPs identified by GRS optimization exert their impact on the development and progression of T2DM mainly through the impairment of pancreatic beta cell functions. For more details on the individual effect of SNPs inserted into the optimized GRS on the development of T2DM see Table S3.

### 3.5. Effects of Genetic (Unweighted Genetic Risk Score) and Non-Genetic (Sex, BMI, TG/HDL-C and Duration of T2DM) Factors on the Age of Onset for T2DM in the Case Population

The mean value of GRS was 7.72 (7.55–7.88) in the full Case population; 7.75 (7.49–8.00) for males and 7.69 (7.46–7.91) for the females. The GRS showed a significant association with the age of onset for T2DM in the full Case population and also separately in both sexes. The TG/HDL-C ratio significantly determined the age of onset for T2DM in the male population (beta = −0.556, *p* < 0.001), while it was not observed in the female one (beta = −0.136, *p* = 0.251). Females are more protected against the early manifestation of T2DM compared to males (males vs. females: beta = 2.352, *p* < 0.001). For more details see Table 2.

There is a significant trend between GRS and the age of onset groups for T2DM in the total Case population and also by sex. The development of T2DM occurred at a younger age among individuals with higher GRS values. For more details see Table 3.

### 3.6. Association of Unweighted Genetic Risk Score with the Present of T2DM in the Hungarian General Population

The GRS did not show a significant association with the presence of type 2 diabetes based on the results of the adjusted logistic regression model in the HG population. All conventional risk factors (age, sex, BMI, and TG/HDL-C ratio) in the model showed a significant correlation with the outcome. See more details in Table 4.

### 3.7. Association of Unweighted Genetic Risk Score with the Age of People in the Hungarian General Population by T2DM Subpopulations

The association of GRS on age was tested by adjusted linear regression models on the subpopulations (based on fasting glucose levels and special treatment for diabetes) of the HG one. A significant correlation between patients’ age and GRS (beta = −0.999, *p* = 0.003) was only measurable in the subpopulation with T2DM (fasting blood glucose levels of 7/L or higher and/or under antidiabetic treatment). Sex showed a significant association with age in the subpopulation with normal glucose levels. BMI showed a significant association with the age of patients in the subpopulation with normal glucose levels and prediabetes. TG/HDL-C ratio had no significant effect in any of the subpopulations. For more detailed results see Table 5.

Five categories were formed based on the GRS (GRS < 4, = 4, = 6, = 8, and > 8). Between the average age of the samples and GRS categories, a significant trend was found only in the subpopulation with T2DM. More detailed in Table S4.

### 3.8. Estimation of the Age of Onset for T2DM by a Score Based On Genetic and Non-Genetic Factors in the Hungarian General Population

An age of onset risk score (AORS) for T2DM was calculated based on the individuals’ sex, BMI, TG/HDL-C ratio, and GRS by multiplying these components with their effects measured on the Case population (see more details in Table 2. A), to estimate the age of onset for T2DM in the HG population.

**Figure 3:**
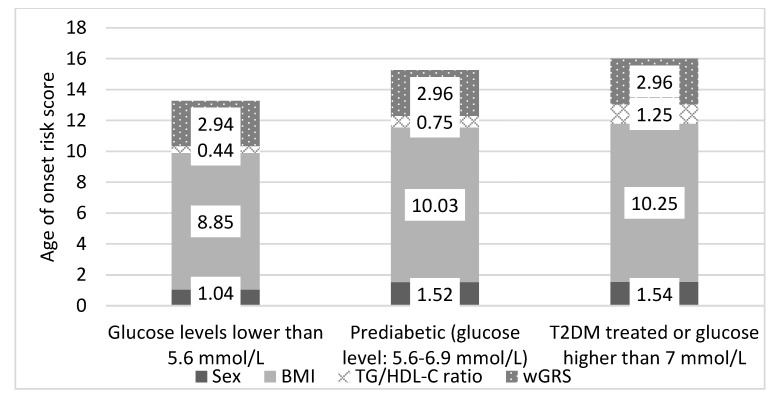
Age of onset risk score values per component (sex, BMI, TG/HDL-C ratio, and wGRS) in the Hungarian general population by subpopulations.

The mean AORS values (normal glucose level: 13.26 vs. prediabetes: 15.27 and T2DM: 16.00) showed a significant difference compared to the samples with prediabetes or T2DM and subpopulation with normal glucose levels. In terms of mean values, all non-genetic components (sex, BMI, and TG/HDL-C ratio) differed at a statistically significant level (*p* < 0.05) between the subpopulation with normal glucose levels and prediabetic or Type 2 diabetic patients. The mean values of wGRS did not differ significantly between the study subpopulations (see more details in Figure 3). This result is consistent with the fact that genetic determination of the age of onset for T2DM is not changeable, it remains constant from birth. Environmental and lifestyle characteristics play a significant role in the development of type 2 diabetes, which is also supported by data in the literature used [7,42,59].

**Figure 4:**
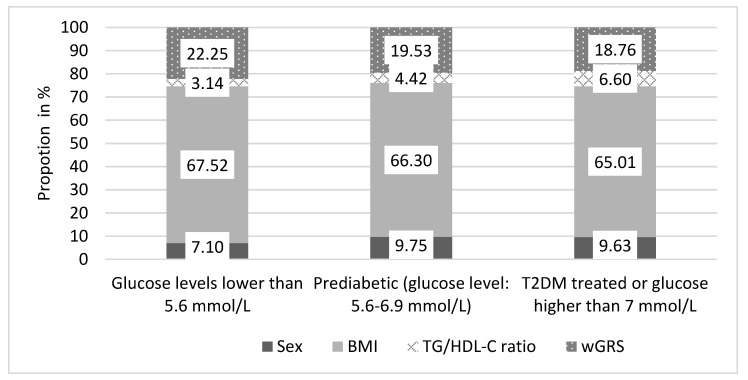
The proportion of components (sex, BMI, TG/HDL-C ratio, and wGRS) of the age of onset risk score in the Hungarian general population by subpopulations.

The representation of AORS’s components (%) in the subpopulations was examined. There is a statistically significant trend (*p* < 0.05) in terms of the proportion of sex, BMI, TG/HDL-C ratio, and wGRS across the subpopulations. In case of sex, the proportion of AORS is higher in the prediabetic and T2DM groups compared to the normal one, which can be explained by a higher proportion of men in the T2DM group. In case of the TG/HDL-C ratio, the proportion of AORS is higher in the prediabetic and T2DM groups than in the normal one, the elevation trend can be explained by the fact that lipid and glucose metabolism are closely related and the TG/HDL-C ratio has been linked to the onset of diabetes based on previous publication [38]. The “weight” of the non-genetic factors is increasing with the progression of T2DM; however, since the genetic component never changes, there is a decreasing trend in the share of genetic risk factors (see more details in Figure 4).

### 3.9. The Effect of wGRS on the Age of Onset for T2DM in the Hungarian General Population

Linear regression analyses were performed to examine the effect of AORS’s components on the age of onset for T2DM on type 2 diabetic subpopulation in the HG one. Out of the four inserted components (sex, BMI, TG/HDL-C ratio, and wGRS), only wGRS showed a significant (*p* = 0.0036) association with the age of onset for T2DM. A one-unit increase in wGRS results in developing T2DM in the studied groups two years earlier, (see more details in Table 6) which shows a striking resemblance to the findings of the study carried out by Zhou et al. on the European population [47].

To describe the association between the wGRS and the age of onset for T2DM, we examined the proportion of wGRS in AORS in three different age groups (≤49 yrs, 50–59 yrs, and ≥60 yrs) in the type 2 diabetic subgroup.

**Figure 5:**
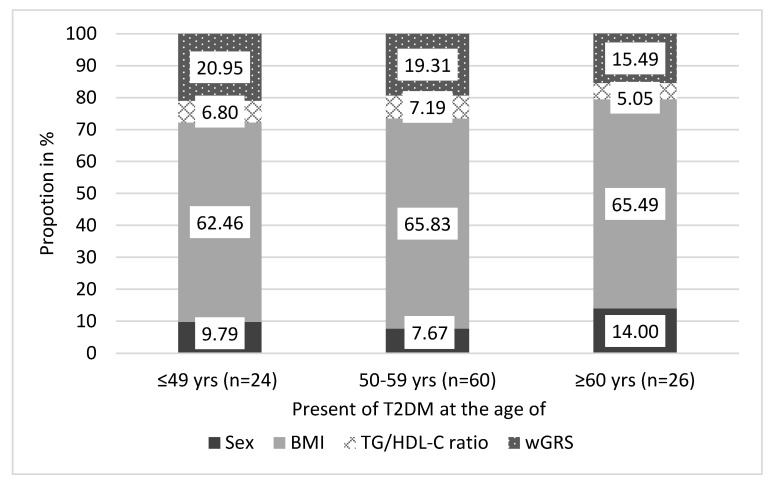
The proportion of components (sex, BMI, TG/HDL-C ratio, and wGRS) of Table 2. diabetic subgroups of the Hungarian general population by age groups.

The trend of wGRS’ proportion increased significantly (*p* = 0.023) from the under 50 years (20.95%) through the 50–59 (19.31%) to the over 60 years of age type 2 diabetic patients (15.49%). The same trend was observed in case of the proportion of sex (≤49 yrs: 9.79%, 50–59 yrs: 7.67%, ≥60 yrs: 14.00%; *p* = 0.016) while in case of the proportion of BMI (*p* = 0.383) and TG/HDL-C ratio (*p* = 0.365) no significant trend was observed across the age groups (see more details in Figure 5).

## 4. Discussion

Over the past few decades, the prevalence of EOT2DM has been increasing throughout the world and it is becoming the predominant form of diabetes among adolescents and younger adults in some populations [7]. Since the early onset of T2DM is associated with a higher cardiovascular risk (micro- and macrovascular complications) and a higher frequency of comorbidities among the patients, early identification of EOT2DM risk would be important for the development of effective preventive intervention strategies. Developing a sensitive risk assessment tool is essential to know the weight of genetic and non-genetic factors in the disease manifestation.

The current study aims to evaluate the joint effect of type 2 diabetes mellitus associated SNPs (using genetic risk score modeling) and known non-genetic risk factors (such as sex, BMI, and TG/HDL-C ratio) on the age of onset for T2DM in the Hungarian general population.

When the effect of SNPs on the age of onset for type 2 diabetes mellitus was evaluated, no SNP was identified to have an individually significant association with the age of onset for type 2 diabetes mellitus. Under the optimization process of the genetic risk score model, 12 SNPs have been identified that increase the strength of the association between the GRS and the age of onset for T2DM on the Case population. The GRS (summarizing the individual effect of SNPs) shows a strongly significant association with the age of onset for type 2 diabetes mellitus (beta (95%CI): −0.454 (−0.674–−0.234), *p* < 0.001) even after adjusting for relevant covariates (sex, BMI, TG/HDL-C and duration of T2DM) in the Case population. The result was similar when this association was evaluated separately for the two sexes (males: beta (95%CI): −0.434 (−0.722–−0.145), *p* = 0.003; females: beta (95%CI): −0.405 (−0.796–−0.120), *p* = 0.008). A significant trend could also be observed between age groups (≤49 yrs, 50–59 yrs, and ≥60 yrs) and the mean value of GRS in the Case population (*p* < 0.001) and separately by sex (males: *p* = 0.002, females: *p* = 0.0038).

We tested the optimized GRS model (created on the Case population) on the HG population. The GRS showed no significant association with the presence of T2DM under the adjusted logistic regression model (OR (95%CI): 1.032 (0.945–1.126), *p* = 0.488). There was no significant association between individual age and GRS in the normal blood glucose (beta (95%CI): 0.104 (−0.158–0.365), *p* = 0.437) and prediabetic groups (beta (95%CI): −0.245 (−0.972–0.483), *p* = 0.507). In case of the type 2 diabetic group, GRS had a significant effect on the age of onset for T2DM; one point increase in AORS point of GRS reduced the age of onset by the average of one year (beta (95%CI): −0.999 (−1.660–−0.337), *p* = 0.003).

A limited number of studies examined and reported the association between genetic risk scores and the age of onset for T2DM. These studies support our findings according to which genetic factors have an effect on the age of onset for T2DM. Iwata M. et al. [43] constructed GRS using 14 SNPs and evaluated its association with the early onset of T2DM in the Japanese population. The same result was observed by Kong X. et al. [31] based on 24 SNPs in the Chinese. A study from pooled data of the Framingham Offspring Study which examined GRS constructed from 40 SNPs on individuals below or above 50 years of age witnessed that the risk per risk allele was higher (more than double) in individuals below 50 years than in people above 50 years of age [60].

The investigators also concluded that the genetic risk score effectively increased the predictive value in addition to clinical risk factors in younger adults (<50 years of age) but not in older people (≥50 years of age). Our findings were also supported by The EPIC InterAct case-cohort study [61] where the relative genetic risk comprised of 49 SNPs is higher in individuals who developed T2DM at a younger age (<55years) compared to that of individuals who developed it later (55–65 years or ≥65 years of age). Similarly, a more recent study by Mars et al. [5], which showed that an individual with higher GRS developed the disease at an earlier age than individuals with lower GRS, concluded that a higher GRS has an impact on the age of onset for T2DM.

In our validation study, we could show that the age of onset was significantly associated with GRS and wGRS among individuals with T2DM. We also show that the higher the GRS is, the lower the age of onset for type 2 diabetes mellitus is (GRS: beta = −0.999, *p* = 0.003; wGRS: beta = −2.011, *p* = 0.0036). To estimate the age of onset for type 2 diabetes mellitus, we calculated the AORS for HG population’s normal, prediabetic, and T2DM subpopulations and found that the risk score was significantly higher in the T2DM group compared to the normal one. In this analysis, significant differences were observed for all non-genetic factors but not for GRS among the three groups (normal, prediabetic, and T2DM). This proves that genetic determination always remains constant throughout the life-course of an individual. A person’s increased genetic risk of the early onset of T2DM only exists if the effects of non-genetic risk factors are high enough. We further analyzed the effect of AORS and its components only for T2DM individuals from the HG population, and the findings explained the association between the age of onset for T2DM and genetic risk scores, i.e., the earlier the age of onset for T2DM is, the higher the GRS and wGRS are.

We observed a very strong association between TG/HDL-C and the age of onset for type 2 diabetes mellitus in men, and no association was seen in women. The age of onset for T2DM could be influenced by the TG/HDL-C ratio in men. In the Bogalusa Heart Study, the investigators observed that the TG/HDL-C ratio was associated with the age of onset for type 2 diabetes mellitus [38]. However, the researchers did not assess their association separately for the male and female populations. Indeed, men are generally diagnosed with diabetes at a younger age than women [62].

The strength of our current study is that we validated our results obtained on a T2DM case group by using an independent sample population (Hungarian general one). It is undeniable that this study has some limitations. Owing to a lack of information on gene-gene interactions, gene-environment interactions, epigenetic factors, and structural variants, we did not consider them in our analysis. In the current study, 12 SNPs that play a role in the age of onset for type 2 diabetes mellitus were considered in the GRS model. Incorporating a larger number of SNPs may further improve the predictive ability of the GRS model. Nonetheless, adding a large number of SNPs into the GRS model does not necessarily lead to a better predictive ability [63,64].

The clinical applicability of our present study is limited, further studies are needed to understand the SNP-mediated development of T2DM. It is also necessary to test the results in other populations to gain certainty that the results obtained are similar in non-Hungarian populations. Nevertheless, our results may pave the way for the development of genetic tests that can be used to predict the timing of T2DM development and delay or prevent its manifestation through targeted interventions, which would also reduce the burden on health care systems.

Owing to the emergence of “big data”, and the advancement of analytical tools based on the results of genomic, epigenetic, metabolomic, proteomic and pharmacogenetic studies, personalized type 2 diabetes treatments are creating, and a one-size-fits-all approach is becoming antiquated. Because of polygenic nature of the disease and the influence of both environmental and genetic factors on its development, defining subgroups using molecular testing is difficult in type 2 diabetes mellitus patients. So the best approach for the accurate and most convenient treatment of T2DM is to categorize patients based on their SNPs-based expected response to medicines. Exploring how the SNPs influence drug efficacy may help us uncover new drug targets and personalized treatment.

In summary, this is the first study that investigated the association of the combined effect of T2DM associated SNPs using GRS on the age of onset for T2DM in the Hungarian population. Genetic risk score modeling showed that the cumulative effect of T2DM related SNPs was associated with the age of onset for type 2 diabetes mellitus. Individuals who developed T2DM at a younger age carried greater risk allele load compared with individuals who developed T2DM later in their life. Our results suggest that there is a considerable genetic predisposition for the early onset of T2DM. Therefore, GRS can be used as a tool for stratifying and estimating the risk of the earlier onset of type 2 diabetes mellitus in the Hungarian population.

## Figures and Tables

**Table 1:** Characteristics of the T2DM case and the Hungarian general population by three categories.

	T2DM Case Population (*n* = 881)	Hungarian General Population			
		Normal FG Levels (FG < 5.6 mmol/L, *n* = 1197)	Prediabetes (FG: 5.6–6.9 mmol/L, *n* = 108)	T2DM (FG ≥ 7 mmol/L and/or Treated, *n* = 110)	*p* for Trend
Male in % (95%CI) ^a^	49.3 (46.0–52.6)	44.0 * (41.2–46.9)	64.8 * (55.5–73.3)	65.5 * (56.3–73.8)	<0.001
Female in % (95%CI) ^a^	50.7 (47.4–54.0)	56.0 * (53.1–58.8)	35.2 * (26.7–44.5)	34.5 * (26.2–43.7)	
Avg. age in years (95%) ^a^	66.14 (65.53–66.74)	42.68 ** (41.99–43.37)	50.57 ** (48.77–52.38)	54.33 ** (52.87–55.79)	<0.001
Avg. BMI in kg/m^2^ (95%)	31.33 (30.97–31.69)	26.81 ** (26.53–27.10)	30.40 (29.17–31.64)	31.06 (29.95–32.17)	<0.001
Avg. HDL-C levels in mmol/L (95%)	1.26 (1.24–1.29)	1.45 ** (1.42–1.47)	1.38 * (1.27–1.48)	1.19 (1.12–1.27)	<0.001
Avg. TG levels in mmol/L (95%)	2.52 (2.36–2.69)	1.47 ** (1.41–1.53)	2.01 (1.64–2.39)	3.13 (2.36–3.89)	<0.001
Avg. TG/HDL-C ratio (95%CI)	2.34 (2.12–2.56)	1.24 ** (1.16–1.33)	2.12 (1.37–2.87)	3.54 (2.34–4.75)	<0.001
Avg. age at diagnosis of T2DM in years (95%CI)	57.25 (56.6–57.9)	-	-	unknown	-

Notes: ^a^ The age and gender differences between the Case and the Hungarian T2DM subpopulations are due to the age category (20–64 years) applied in the sample collection of the Hungarian general population. CI: confidence interval. * statistically significant (*p* < 0.05) difference from T2DM case population. ** Bonferroni corrected significant (*p* < 0.0042) difference from T2DM case population.

**Table 2:** Association of genetic risk scores (GRS) with the age of onset for T2DM by full Case population (A) and separate for sex (B in males, C in females). The association was evaluated under adjusted regression models (sex, BMI, TG/HDL-C ratio).

**A- Full Population**	**Beta (95%CI)**	***p*-Value**
Sex	2.352 (1.228–3.475)	<0.001 **
BMI	−0.330 (−0.434–−0.227)	<0.001 **
TG/HDL-C ratio	−0.354 (−0.511–−0.198)	<0.001 **
Duration of T2DM	−0.607 (−0.698–−0.515)	<0.001 **
GRS	−0.454 (−0.674–−0.234)	<0.001 **
**B- Males**	**Beta (95%CI)**	***p*-value**
BMI	−0.290 (−0.435–−0.145)	<0.001 **
TG/HDL-C ratio	−0.556 (−0.767–−0.346)	<0.001 **
Duration of T2DM	−0.646 (−0.774–−0.517)	<0.001 **
GRS	−0.434 (−0.722–−0.145)	0.003 **
**C- Females**	**Beta (95%CI)**	***p*-value**
BMI	−0.376 (−0.523–−0.229)	<0.001 **
TG/HDL-C ratio	−0.136 (−0.369–0.097)	0.251
Duration of T2DM	−0.579 (−0.708–−0.450)	<0.001 **
GRS	−0.405 (−0.796–−0.120)	0.008 *

Notes: CI: confidence interval. * statistically significant *p*-value (*p* < 0.05). ** significant *p*-value with Bonferroni correction

**Table 3:** The average value of GRS in the age categories of onset for T2DM and the results of *p* for trend analyses in the T2DM case population.

	≤49 years	50–59 years	≥60 years	*p* for Trend
	Mean GRS (95%CI)	Mean GRS (95%CI)	Mean GRS (95%CI)	
Full population	8.36 (7.97–8.75, *n* = 191)	7.79 ^a^ (7.52–8.06, *n* = 340)	7.30 ^b^ (7.04–7.55, *n* = 350)	<0.001 **
Males	8.18 (7.63–8.73, *n* = 111)	8.00 (7.61–8.39, *n* = 176)	7.12 ^b^ (6.71–7.52, *n* = 147)	0.002 **
Females	8.60 (8.05–9.15, *n* = 80)	7.56 ^b^ (7.20–7.92, *n* = 164)	7.43 ^b^ (7.10–7.76, *n* = 203)	0.0038 **

Notes: ^a^ statistically significant difference (*p* < 0.05) compared with the ≤49 years old T2DM subpopulation. ^b^ significant *p*-value with Bonferroni correction compared with the ≤49 years old T2DM subpopulation. CI: confidence interval. ** significant *p*-value with Bonferroni correction.

**Table 4:** Association of genetic risk scores (GRS) with T2DM status (person with fasting glucose levels of less than 5.6 mmol/L vs. fasting blood glucose levels of 7 mmol/L or higher and/or was under antidiabetic treatment) in the Hungarian general population. The association was evaluated under an adjusted regression model (by age, sex, BMI, and TG/HDL-C ratio).

	OR (95%CI)	*p*-Value
Age	1.087 (1.063–1.112)	<0.001 **
Sex	0.502 (0.320–0.788)	0.003 **
BMI	1.066 (1.025–1.109)	0.001 **
TG/HDL-C ratio	1.312 (1.186–1.451)	<0.001 **
GRS	1.032 (0.945–1.126)	0.488

Notes: CI: confidence interval. ** statistically significant *p*-value with Bonferroni correction.

**Table 5:** Association of genetic risk scores (GRS) with age in subpopulations for T2DM (*n* = 110), prediabetes (*n* = 108), and people with normal glucose levels (*n* = 1197). The association was evaluated by using adjusted linear regression models (sex, BMI, and TG/HDL-C ratio).

**Normal Glucose**	**Beta (95%CI)**	***p*-Value**
Sex	3.293 (1.991–4.596)	<0.001 **
BMI	0.621 (0.510–0.732)	<0.001 **
TG/HDL-C ratio	0.088 (−0.213–0.388)	0.567
GRS	0.104 (−0.158–0.365)	0.437
**Prediabetes**	**Beta (95%CI)**	***p*-Value**
Sex	1.559 (−2.358–5.476)	0.432
BMI	0.330 (0.024–0.635)	0.035 *
TG/HDL-C ratio	−0.338 (−1.092–0.416)	0.376
GRS	−0.245 (−0.972–0.483)	0.507
**T2DM**	**Beta (95%CI)**	***p*-Value**
Sex	0.128 (−3.296–3.553)	0.941
BMI	0.165 (−0.086–0.416)	0.195
TG/HDL-C ratio	−0.413 (−1.088–0.262)	0.228
GRS	−0.999 (−1.660–−0.337)	0.003 **

Notes: CI: confidence interval. * statistically significant *p*-value (*p* < 0.05). ** statistically significant *p*-value with Bonferroni correction.

**Table 6:** Results of the linear regression model of AORS’s components on the age of onset for T2DM in the Hungarian general population’s type 2 diabetic subpopulation.

	Beta (95%CI)	*p*-Value
Sex	−0.346 (−1.667–0.975)	0.604
BMI	0.485 (−0.250–1.221)	0.194
TG/HDL-C ratio	−0.167 (−0.803–0.469)	0.605
wGRS	−2.011 (−3.347–−0.674)	0.0036 **

Notes: CI: confidence interval. ** statistically significant *p*-value with Bonferroni correction.

## Data Availability

Data available on request due to restrictions e.g., privacy or ethical.

## Supplementary Materials

The following are available online at https://www.mdpi.com/2075-4426/11/1/6/s1, Table S1: List of SNPs (coded by the most fitting genetic model of inheritance) and their effect on the age of onset of T2DM in the adjusted (by BMI, TG/HDL-C ratio, sex, and duration of T2DM) regression model in order of p-value (from the lowest value to the highest)., Table S2: List of SNPs which were used for the GRS calculation. Table S3: The individual effect of SNPs included in the optimized GRS model on the development and progression of T2DM based on the literature. Table S4: The average age in years by GRS category in the Hungarian general population.
