# Supplementary material for: Impact of Genetic Factors on the Age of Onset for Type 2 Diabetes Mellitus in Addition to the Conventional Risk Factors
Source: J Pers Med. 2020 Dec 22;11(1):6. doi: 10.3390/jpm11010006 (PMC7822179; doi:10.3390/jpm11010006)
Supplement: Supplementary file 1 [file jpm-11-00006-s001.zip › Supplementary_table_1.docx]

**Supplementary** **Table 1.** List of SNPs (coded by the most fitting genetic model of inheritance) and their effect on the age of onset of T2DM in the adjusted (by BMI, TG/HDL-C ratio, sex, and duration of T2DM) regression model in order of p-value (from the lowest value to the highest).

| **No.** | **SNP** | **Gene** | **Effect allele** | **Genetic model** | **Beta (95CI)** | **p-value** |
| --- | --- | --- | --- | --- | --- | --- |
| 1 | rs174550 | FADS1 | C | Recessive | -0.866 (-1.812 - 0.079) | 0.073 |
| 2 | rs7903146 | TCF7L2 | T | Recessive | -0.782 (-1.719 - 0.155) | 0.102 |
| 3 | rs7944584 | MADD | A | Recessive | -0.467 (-1.033 - 0.099) | 0.106 |
| 4 | rs10830963 | MTNR1B | G | Dominant | -0.426 (-0.995 - 0.143) | 0.142 |
| 5 | rs7034200 | GLIS3 | A | Dominant | -0.413 (-1.067 - 0.240) | 0.215 |
| 6 | rs10885122 | ADRA2A | T | Recessive | -1.326 (-3.586 - 0.934) | 0.250 |
| 7 | rs5219 | KCNJ11 | T | Recessive | -0.427 (-1.181 - 0.326) | 0.266 |
| 8 | rs3736594 | MRPL33 | C | Recessive | -0.575 (-1.695 - 0.545) | 0.314 |
| 9 | rs560887 | G6PC2 | T | Recessive | -0.479 (-1.472 - 0.512) | 0.344 |
| 10 | rs11671664 | GIPR | G | Recessive | -0.272 (-0.932 - 0.388) | 0.419 |
| 11 | rs10946398 | CDKAL1 | C | Codominant | -0.322 (-1.168 - 0.524) | 0.455 |
| 12 | rs11920090 | SLC2A2 | T | Recessive | -0.242 (-0.901 - 0.416) | 0.470 |
| 13 | rs7173964 | C2CD4B | G | Recessive | -0.200 (-0.795 - 0.394) | 0.509 |
| 14 | rs10811661 | CDKN2A/B | C | Recessive | -0.573 (-2.437 - 1.291) | 0.546 |
| 15 | rs340874 | PROX1 | C | Dominant | -0.133 (-0.768 - 0.502) | 0.680 |
| 16 | rs10906115 | CDC123/CAMK1D | G | Dominant | -0.119 (-0.703 - 0.466) | 0.691 |
| 17 | rs11071657 | C2CD4B | G | Dominant | -0.084 (-0.664 - 0.496) | 0.776 |
| 18 | rs780094 | GCKR | C | Recessive | -0.066 (-0.666 - 0.534) | 0.830 |
| 19 | rs1111875 | HHEX | C | Recessive | -0.055 (-0.632 - 0.521) | 0.851 |
| 20 | rs11558471 | SLC30A8 | G | Recessive | -0.087 (-1.106 - 0.931) | 0.866 |
| 21 | rs2191349 | DGKB | T | Recessive | -0.004 (-0.596 - 0.589) | 0.990 |

CI: confidence interval
