# Supplementary material for: Impact of Genetic Factors on the Age of Onset for Type 2 Diabetes Mellitus in Addition to the Conventional Risk Factors
Source: J Pers Med. 2020 Dec 22;11(1):6. doi: 10.3390/jpm11010006 (PMC7822179; doi:10.3390/jpm11010006)
Supplement: Supplementary file 1 [file jpm-11-00006-s001.zip › Supplementary_table_2.docx]

**Supplementary Table 2.** List of SNPs which were used for the GRS calculation

| **Steps** | **Inserted SNP** | **GRS association** | | | **Included/excluded from the GRS** |
| --- | --- | --- | --- | --- | --- |
|  |  | **beta** | **p-value** | **R-square** |  |
| 1 | rs174550 | -0.866 (-1.812 – 0.079) | 0.073 | 0.228 ↑ | Included |
| 2 | rs7903146 | -0.865 (-1.546 - -0.184) | 0.013 | 0.230 ↑ | Included |
| 3 | rs7944584 | -0.627 (-1.060 - -0.193) | 0.005 | 0.232 ↑ | Included |
| 4 | rs10830963 | -0.533 (-0.871 - -0.195) | 0.002 | 0.233 ↑ | Included |
| 5 | rs7034200 | -0.510 (-0.810 - -0.210) | 9x10^-4^ | 0.235 ↑ | Included |
| 6 | rs10885122 | -0.527 (-0.825 - -0.229) | 6x10^-4^ | 0.235 ↑ | Included |
| 7 | rs5219 | -0.510 (-0.785 - -0.234) | 3x10^-4^ | 0.236 ↑ | Included |
| 8 | rs3736594 | -0.526 (-0.797 - -0.255) | 1.5x10^-4^ | 0.238 ↑ | Included |
| 9 | rs560887 | -0.517 (-0.777 - -0.258) | 1x10^-4^ | 0.238 ↑ | Included |
| 10 | rs11671664 | -0.478 (-0.713 - -0.243) | 7x10^-5^ | 0.239 ↑ | Included |
| 11 | rs10946398 | -0.463 (-0.713 - -0.243) | 9x10^-5^ | 0.238 ↓ | Excluded |
| 12 | rs11920090 | -0.456 (-0.679 - -0.234) | 6x10^-5^ | 0.239 ↑ | Included |
| 13 | rs7173964 | -0.410 (-0.615 - -0.205) | 9x10^-5^ | 0.238 ↓ | Excluded |
| 14 | rs10811661 | -0.454 (-0.674 - -0.234) | 5.5x10^-5^ | 0.239 ↑ | Included |
| 15 | rs340874 | -0.420 (-0.628 - -0.212) | 8x10^-5^ | 0.238 ↓ | Excluded |
| 16 | rs10906115 | -0.387 (-0.587 - -0.187) | 1.5x10^-4^ | 0.238 ↓ | Excluded |
| 17 | rs11071657 | -0.415 (-0.623 - -0.208) | 9x10^-4^ | 0.238 ↓ | Excluded |
| 18 | rs780094 | -0.392 (-0.595 - -0.190) | 1.5x10^-4^ | 0.238 ↓ | Excluded |
| 19 | rs1111875 | -0.386 8-0.591 - -0.181) | 2x10^-4^ | 0.237 ↓ | Excluded |
| 20 | rs11558471 | -0.434 (-0.648 - -0.220) | 8x10^-5^ | 0.238 ↓ | Excluded |
| 21 | rs2191349 | -0.403 (-0.610 - -0.195) | 1.5x10^-4^ | 0.238 ↓ | Excluded |

SNPs with improving effect on correlation shadowed

CI: confidence interval
