# Supplementary material for: Impact of Genetic Factors on the Age of Onset for Type 2 Diabetes Mellitus in Addition to the Conventional Risk Factors
Source: J Pers Med. 2020 Dec 22;11(1):6. doi: 10.3390/jpm11010006 (PMC7822179; doi:10.3390/jpm11010006)
Supplement: Supplementary file 1 [file jpm-11-00006-s001.zip › Supplementary_table_3.docx]

**Supplementary Table 3.** The individual effect of SNPs included in the optimized GRS model on the development and progression of T2DM based on the literature

| SNP | Gene | Effect on T2DM disease procession |
| --- | --- | --- |
| rs174550 | FADS1 | ADS1 encodes rate limiting enzyme known as delta-5 desaturase (D5D). D5D is responsible for the double bond formation in the n-3 poly unsaturated fatty acid (PUFA) pathway and is linked with fatty acid composition in plasma, adipose tissue and membrane fluidity [1]. Mutation rs174550 mediate the development of type 2 diabetes mellitus by impairing insulin sensitivity [2,3]. |
| rs7903146 | TCF7L2 | TCF7L2 encodes a high mobility group box-containing transcription factor which involved in Wnt signaling pathway[4]. This Wnt signaling pathway plays significant role in the islet cell proliferation and differentiation in the pancreas. The rs7903146 is associated with impaired beta-cell function [5] and reduced insulin secretion [6] but not with insulin resistance and enhance the rate of hepatic glucose production [7]. The rs7903146 polymorphism subvert the Wnt signaling pathway and impedes the insulin secretion, and finally ends up with progression of type 2 diabetes mellitus [8,9]. |
| rs7944584 | MADD | The biological function of MADD is linked with pancreatic beta cell proliferation and development [10]. It encodes mitogen-activated protein kinase (MAPK) activating death domain, an adaptor protein that interacts with the tumor necrosis factor alpha receptor to activate MAPK. MAPK is believed to be involved in the proliferation of pancreatic beta cells and insinuating that rs7944584 polymorphism plays crucial progression of T2DM through beta cell dysfunctions [11]. |
| rs10830963 | MTNR1B | MTNR1B encodes the melatonin receptor MT2, a G protein-coupled receptor, which is expressed in pancreatic islets [12]. The rs10830963 associated with higher fasting glucose levels and lower dynamic beta cell response [13] and increased the risk of isolated impaired fasting glycemia but not isolated impaired glucose tolerance [14]. |
| rs7034200 | GLIS3 | GLIS3 plays a key role in controlling insulin gene transcription, insulin secretion and pancreatic beta cell survival. The rs7034200 is associated with fasting glucose and impaired β cell function [15,16] and associated with reduced glucose-stimulated β cell function [17]. |
| rs10885122 | ADRA2A | ADRA2A encodes the alpha2A-adrenergic receptor (alpha(2A)AR), a Gi-coupled receptor expressed in pancreatic beta cells and whose activation leads to an outward potassium current independent of the islet ATP-sensitive potassium channel. By this way they modify the release of insulin. The rs10885122 polymorphism mediates adrenergic suppression of insulin secretion, and in turn increase the development of T2DM [18]. |
| rs5219 | KCNJ11 | KCNJ11 encodes Kir6.2 protein that forms the inner section of the adenosine triphosphate sensitive potassium ion channel (KATP) in the pancreatic beta cells and plays important role in insulin secretion. The rs5219 can affect the insulin secretion pathway. The risk allele (A) of this locus impairs this pathway by reducing ATP sensitivity of the KATP channel, hence resulting in over activity of the channel and subsequent suppression of insulin secretion. This effect on insulin secretion is more significant in carriers of the AA genotype compared with carriers of the GA genotype [19]. The A allele carriers have higher HbA1c levels [20], higher blood pressure [20], better therapeutic response to gliclazide [21] and sulfonylurea therapy [22] than the G allele carriers. |
| rs3736594 | MRPL33 | Mitochondrial ribosomal protein L33 gene encodes a large mitoribosomal subunit protein, which may be involved in mitochondrial translation. The rs3736594 associated with fasting glucose and insulin levels [23]. |
| rs560887 | G6PC2 | G6PC2 encodes the enzyme islet- specific glucose -6-phosphoatse catalytic subunit related protein (IGRP) that takes part in the counter player to glucokinase by dephosphorylating glucose-6-phosphate and ends up with glucose stimulated insulin secretion, thus mutation in the rs560887 leads to the development of T2DM [24]. |
| rs11671664 | GIPR | GIPR is expressed in pancreatic islets and adipocytes, and linked with insulin resistance and T2DM, stimulation of glucose-stimulated insulin secretion, modulation of beta cell neogenesis and pancreatic beta cell differentiation and proliferation [25] and it is thought that mutation in the rs11671664 may results in T2DM. |
| rs11920090 | SLC2A2 | SLC2A2 encodes GLUT2, a glucose transporter and a member of the facilitative glucose transporter family, is highly expressed in pancreatic beta cells and lever. GLUT2 is involved in the regulation of both glucose uptake and output. rs11920090 polymorphism may probably influence basal insulin secretion and mediates the progression of type 2 diabetes [24]. |
| rs10811661 | CDKN2A/B | CDKN2A/B inhibits the activity of CDK4 and CDK6 which involved in the pancreatic beta cell function and regeneration. The rs10811661 polymorphism influences pancreatic beta cell proliferation in pancreatic islets and mass, and further results in the development of diabetes [26]. |

1. Huang, M.-C.; Chang, W.-T.; Chang, H.-Y.; Chung, H.-F.; Chen, F.-P.; Huang, Y.-F.; Hsu, C.-C.; Hwang, S.-J. FADS gene polymorphisms, fatty acid desaturase activities, and HDL-C in type 2 diabetes. *International Journal of Environmental Research and Public Health* **2017**, *14*, 572.

2. Warensjö, E.; Rosell, M.; Hellenius, M.-L.; Vessby, B.; De Faire, U.; Risérus, U. Associations between estimated fatty acid desaturase activities in serum lipids and adipose tissue in humans: links to obesity and insulin resistance. *Lipids in health and disease* **2009**, *8*, 37.

3. Cormier, H.; Rudkowska, I.; Thifault, E.; Lemieux, S.; Couture, P.; Vohl, M.-C. Polymorphisms in Fatty Acid Desaturase (FADS) gene cluster: Effects on glycemic controls following an omega-3 Polyunsaturated Fatty Acids (PUFA) supplementation. *Genes* **2013**, *4*, 485-498.

4. Lyssenko, V.; Lupi, R.; Marchetti, P.; Del Guerra, S.; Orho-Melander, M.; Almgren, P.; Sjögren, M.; Ling, C.; Eriksson, K.-F.; Mancarella, R. Mechanisms by which common variants in the TCF7L2 gene increase risk of type 2 diabetes. *The Journal of clinical investigation* **2007**, *117*, 2155-2163.

5. Florez, J.C.; Jablonski, K.A.; Bayley, N.; Pollin, T.I.; de Bakker, P.I.; Shuldiner, A.R.; Knowler, W.C.; Nathan, D.M.; Altshuler, D.; Diabetes Prevention Program Research, G. TCF7L2 polymorphisms and progression to diabetes in the Diabetes Prevention Program. *N Engl J Med* **2006**, *355*, 241-250, doi:10.1056/NEJMoa062418.

6. Palmer, N.D.; Lehtinen, A.B.; Langefeld, C.D.; Campbell, J.K.; Haffner, S.M.; Norris, J.M.; Bergman, R.N.; Goodarzi, M.O.; Rotter, J.I.; Bowden, D.W. Association of TCF7L2 gene polymorphisms with reduced acute insulin response in Hispanic Americans. *J Clin Endocrinol Metab* **2008**, *93*, 304-309, doi:10.1210/jc.2007-1225.

7. Lyssenko, V.; Lupi, R.; Marchetti, P.; Del Guerra, S.; Orho-Melander, M.; Almgren, P.; Sjogren, M.; Ling, C.; Eriksson, K.F.; Lethagen, A.L., et al. Mechanisms by which common variants in the TCF7L2 gene increase risk of type 2 diabetes. *J Clin Invest* **2007**, *117*, 2155-2163, doi:10.1172/JCI30706.

8. Villareal, D.T.; Robertson, H.; Bell, G.I.; Patterson, B.W.; Tran, H.; Wice, B.; Polonsky, K.S. TCF7L2 variant rs7903146 affects the risk of type 2 diabetes by modulating incretin action. *diabetes* **2010**, *59*, 479-485.

9. Huang, Z.-q.; Liao, Y.-q.; Huang, R.-z.; Chen, J.-p.; Sun, H.-l. Possible role of TCF7L2 in the pathogenesis of type 2 diabetes mellitus. *Biotechnology & Biotechnological Equipment* **2018**, *32*, 830-834.

10. Li, L.-c.; Wang, Y.; Carr, R.; Haddad, C.S.; Li, Z.; Qian, L.; Oberholzer, J.; Maker, A.V.; Wang, Q.; Prabhakar, B.S. IG20/MADD plays a critical role in glucose-induced insulin secretion. *Diabetes* **2014**, *63*, 1612-1623.

11. Dupuis, J.; Langenberg, C.; Prokopenko, I.; Saxena, R.; Soranzo, N.; Jackson, A.U.; Wheeler, E.; Glazer, N.L.; Bouatia-Naji, N.; Gloyn, A.L. New genetic loci implicated in fasting glucose homeostasis and their impact on type 2 diabetes risk. *Nature genetics* **2010**, *42*, 105-116.

12. Yin, X. MTNR1B gene polymorphisms are associated with the therapeutic responses to repaglinide in Chinese patients with type 2 diabetes mellitus. *Frontiers in pharmacology* **2019**, *10*, 1318.

13. Zheng, C.; Dalla Man, C.; Cobelli, C.; Groop, L.; Zhao, H.; Bale, A.E.; Shaw, M.; Duran, E.; Pierpont, B.; Caprio, S., et al. A common variant in the MTNR1b gene is associated with increased risk of impaired fasting glucose (IFG) in youth with obesity. *Obesity (Silver Spring)* **2015**, *23*, 1022-1029, doi:10.1002/oby.21030.

14. Sparso, T.; Bonnefond, A.; Andersson, E.; Bouatia-Naji, N.; Holmkvist, J.; Wegner, L.; Grarup, N.; Gjesing, A.P.; Banasik, K.; Cavalcanti-Proenca, C., et al. G-allele of intronic rs10830963 in MTNR1B confers increased risk of impaired fasting glycemia and type 2 diabetes through an impaired glucose-stimulated insulin release: studies involving 19,605 Europeans. *Diabetes* **2009**, *58*, 1450-1456, doi:10.2337/db08-1660.

15. Hong, K.W.; Chung, M.; Cho, S.B. Meta-analysis of genome-wide association study of homeostasis model assessment beta cell function and insulin resistance in an East Asian population and the European results. *Mol Genet Genomics* **2014**, *289*, 1247-1255, doi:10.1007/s00438-014-0885-6.

16. Barrett, J.C.; Clayton, D.G.; Concannon, P.; Akolkar, B.; Cooper, J.D.; Erlich, H.A.; Julier, C.; Morahan, G.; Nerup, J.; Nierras, C., et al. Genome-wide association study and meta-analysis find that over 40 loci affect risk of type 1 diabetes. *Nat Genet* **2009**, *41*, 703-707, doi:10.1038/ng.381.

17. Boesgaard, T.W.; Grarup, N.; Jorgensen, T.; Borch-Johnsen, K.; Meta-Analysis of, G.; Insulin-Related Trait, C.; Hansen, T.; Pedersen, O. Variants at DGKB/TMEM195, ADRA2A, GLIS3 and C2CD4B loci are associated with reduced glucose-stimulated beta cell function in middle-aged Danish people. *Diabetologia* **2010**, *53*, 1647-1655, doi:10.1007/s00125-010-1753-5.

18. Rosengren, A.H.; Jokubka, R.; Tojjar, D.; Granhall, C.; Hansson, O.; Li, D.-Q.; Nagaraj, V.; Reinbothe, T.M.; Tuncel, J.; Eliasson, L. Overexpression of alpha2A-adrenergic receptors contributes to type 2 diabetes. *Science* **2010**, *327*, 217-220.

19. Liu, Z.; Zhang, Y.W.; Feng, Q.P.; Li, Y.F.; Wu, G.D.; Zuo, J.; Xiao, X.H.; Fang, F.D. [Association analysis of 30 type 2 diabetes candidate genes in Chinese Han population]. *Zhongguo Yi Xue Ke Xue Yuan Xue Bao* **2006**, *28*, 124-128.

20. He, Y.Y.; Zhang, R.; Shao, X.Y.; Hu, C.; Wang, C.R.; Lu, J.X.; Bao, Y.Q.; Jia, W.P.; Xiang, K.S. Association of KCNJ11 and ABCC8 genetic polymorphisms with response to repaglinide in Chinese diabetic patients. *Acta Pharmacol Sin* **2008**, *29*, 983-989, doi:10.1111/j.1745-7254.2008.00840.x.

21. Javorsky, M.; Klimcakova, L.; Schroner, Z.; Zidzik, J.; Babjakova, E.; Fabianova, M.; Kozarova, M.; Tkacova, R.; Salagovic, J.; Tkac, I. KCNJ11 gene E23K variant and therapeutic response to sulfonylureas. *Eur J Intern Med* **2012**, *23*, 245-249, doi:10.1016/j.ejim.2011.10.018.

22. Holstein, A.; Hahn, M.; Stumvoll, M.; Kovacs, P. The E23K variant of KCNJ11 and the risk for severe sulfonylurea-induced hypoglycemia in patients with type 2 diabetes. *Horm Metab Res* **2009**, *41*, 387-390, doi:10.1055/s-0029-1192019.

23. Liang, X.; He, A.; Wang, W.; Liu, L.; Du, Y.; Fan, Q.; Li, P.; Wen, Y.; Hao, J.; Guo, X., et al. Integrating Genome-Wide Association and eQTLs Studies Identifies the Genes and Gene Sets Associated with Diabetes. *Biomed Res Int* **2017**, *2017*, 1758636, doi:10.1155/2017/1758636.

24. Heni, M.; Ketterer, C.; Hart, L.M.t.; Ranta, F.; van Haeften, T.W.; Eekhoff, E.M.; Dekker, J.M.; Boomsma, D.I.; Nijpels, G.; Kramer, M.H. The impact of genetic variation in the G6PC2 gene on insulin secretion depends on glycemia. *The Journal of Clinical Endocrinology & Metabolism* **2010**, *95*, E479-E484.

25. Wang, T.; Ma, X.; Tang, T.; Higuchi, K.; Peng, D.; Zhang, R.; Chen, M.; Yan, J.; Wang, S.; Yan, D. The effect of glucose-dependent insulinotropic polypeptide (GIP) variants on visceral fat accumulation in Han Chinese populations. *Nutrition & diabetes* **2017**, *7*, e278-e278.

26. Peng, F.; Hu, D.; Gu, C.; Li, X.; Li, Y.; Jia, N.; Chu, S.; Lin, J.; Niu, W. The relationship between five widely-evaluated variants in CDKN2A/B and CDKAL1 genes and the risk of type 2 diabetes: a meta-analysis. *Gene* **2013**, *531*, 435-443.
