# Supplementary material for: Impact of Genetic Factors on the Age of Onset for Type 2 Diabetes Mellitus in Addition to the Conventional Risk Factors
Source: J Pers Med. 2020 Dec 22;11(1):6. doi: 10.3390/jpm11010006 (PMC7822179; doi:10.3390/jpm11010006)
Supplement: Supplementary file 1 [file jpm-11-00006-s001.zip › Supplementary_table_4.docx]

**Supplementary Table 4.** The average age in years by GRS category in the Hungarian general population.

| No. of risk alleles | **GRS < 4, n=91** | **GRS = 4, n=286** | **GRS = 6, n=469** | **GRS =8, n=379** | **GRS > 8, n=190** | **p for trend** |
| --- | --- | --- | --- | --- | --- | --- |
|  | **Mean age (95%CI)** | | | | |  |
| Normal glucose levels  (GLU<5.6 mmol/l) | 41.68  (38.84 – 44.53) | 43.30  (41.75 – 44.85) | 42.16  (40.96 – 43.35) | 42.50  (41.14 – 43.85) | 43.91  (42.08 – 45.74) | 0.563 |
| Prediabetes  (5.6–6.9 mmol/l) | 49.71  (39.64 – 59.78) | 50.88  (46.00 – 55.75) | 52.20  (48.35 – 56.05) | 49.03  (46.20 – 51.86) | 50.64  (47.11 – 54.18) | 0.222 |
| T2DM  (treated and/or GLU≥7mmol/l) | 58.38  (53.28 – 63.47) | 57.32  (55.73 – 58.90) | 54.81  (52.46 – 57.15) | 52.19^a^  (49.05 – 55.32) | 51.80^a^  (46.11 – 57.49) | 0.0045* |

CI: confidence interval

*statistically significant p-value (p<0.05)

^a^: average age at least nominal significantly differed compared with the GRS<4 subgroup
